# Clinical Profile and Determinants of Mortality in Patients With COVID-19: A Retrospective Analytical Cross-Sectional Study in a Tertiary Care Center in South India

**DOI:** 10.7759/cureus.23103

**Published:** 2022-03-12

**Authors:** Surupa S Kurien, Regi S David, Ajitha K Chellappan, Ravi P Varma, Padmakumar R Pillai, Induprabha Yadev

**Affiliations:** 1 Department of Pathology, Government Medical College & Hospital, Thiruvananthapuram, IND; 2 Department of Internal Medicine, Government Medical College & Hospital, Thiruvananthapuram, IND; 3 Department of Community Medicine, Sree Chitra Tirunal Institute for Medical Sciences and Technology, Thiruvananthapuram, IND; 4 Department of General Surgery, Government Medical College & Hospital, Thiruvananthapuram, IND

**Keywords:** india, absolute neutrophil count, blood urea nitrogen, c-reactive protein, d- dimer, predictors, mortality, covid-19

## Abstract

Introduction

The COVID-19 pandemic gained ground in India, starting from a few cases and spreading to the whole country; eventually becoming the second-most affected country worldwide. Here, we present the clinical and laboratory profile and the risk factors associated with mortality in COVID-19. The study comes from Kerala, a region that reported the first case in India. Kerala has the second-highest case burden in the country but also has managed to keep the case fatality rate down below the national average.

Methodology

This is a single-center retrospective cross-sectional study on 391 laboratory-confirmed COVID-19 positive inpatients between September 2020 and October 2020. Hematological parameters, coagulation parameters, liver function tests (LFT), and renal function tests (RFT) results were collected and compared among survivors and non-survivors to identify predictive biomarkers of mortality.

Results

The mean age of all patients was 53.2 years (SD 17.0). On bivariate analyses, the mean values of total leukocyte count (TLC), absolute neutrophil count (ANC), neutrophil-to-lymphocyte ratio (NLR), C-reactive protein (CRP), ferritin, lactate dehydrogenase (LDH), D-dimer at admission, prothrombin time international normalized ratio (PT INR), blood urea nitrogen (BUN), and creatinine were significantly higher in non-survivors than in survivors: mean (SD) 11.9 (7.6) vs 7.5 (4.2) (x10^9^/L), 10.5 (7.4) vs 5.3 (4.1) (x10^9^/L), 11.6 (13.5) vs 3.4 (3.5), 185 (117) vs 48 (85) (mg/L), 829.4 (551.2) vs 323.6 (374.1) (ng/ml), 905.5 (589.1) vs 485.1 (353.9) (U/L), 4.01 (3.53) vs 1.29 (2.08) (µg/ml), 1.21 (0.42) vs 0.99 (0.18), 105.1 (91.4) vs 33.6 (31.0) (mg/dl), 3.6 (4.1) vs 1.1 (1.6) (mg/dl), respectively, p < 0.001. Absolute lymphocyte count, serum albumin, and albumin/globulin (A/G) ratio were lower in non-survivors than in survivors (mean (SD) 1.3 (1.0) vs 2.0 (0.9) (x10^9^/L), p < 0.001; 3.0 (0.7) vs 3.8 (2.1) (g/dl), p 0.005; 0.9 (0.3) vs 1.2 (0.4), p < 0.001). Multivariate analysis identified ANC, D-dimer at admission, CRP, and BUN as independent prognostic factors associated with mortality.

Conclusion

Several accessible tests like TLC, ANC, NLR, and BUN can be used in low-resource settings to assess severity in patients with COVID-19. In addition, ANC, D-dimer at admission, CRP, and BUN can be used as independent predictors of in-patient mortality in COVID-19 patients in hospital settings.

## Introduction

In December 2019, a cluster of pneumonia cases was reported from Wuhan in Hubei province of China, which was subsequently identified as infection by SARS-CoV-2, a novel β coronavirus of group 2B [1]. On March 11, 2020, WHO characterized the outbreak as a pandemic [2]. India reported its first case on January 27, 2020, from the state of Kerala [3]. As the pandemic ravaged the world, India bore a sizable burden, eventually becoming the nation with the second-highest number of confirmed cases. The state of Kerala that reported the first case in India also became the state with the second-highest number of confirmed cases in the country. Despite Kerala being a region with many odds stacked against it - it has a high density of population (859 persons per km^2^), the highest percentage of geriatric population in the country (16.5% as per the report published by the Ministry of Statistics and Programme Implementation), and a high prevalence of comorbidities - it has nevertheless managed to keep the case fatality rate at 0.96%, which is below the national average, speaking volumes of the better healthcare resources available [4]. In this context, it is important to identify the factors associated with mortality in COVID-19 patients in the local population.

Several factors have emerged as predictive biomarkers of mortality or adverse disease prognosis in various studies conducted mainly outside the Indian subcontinent. Significant among them are neutrophilia, neutrophil-to-lymphocyte ratio (NLR), lymphopenia, inflammatory markers like C-reactive protein (CRP), and cytokines including interleukin-6 (IL-6), interleukin-8 (IL-8), interleukin-18 (IL-18), interferon-α (IFN-α), and tumor necrosis factor (TNF) [5-7]. The rapid innate immunity against COVID-19 is mounted by IFNs, activation of macrophages and neutrophils leading to proinflammatory cytokine production. Cytokine storm is spearheaded by hyperactivation of neutrophils and monocytes [8]. Accordingly, neutrophilia and increased NLR have also emerged as significant biomarkers of disease severity. Qin et al. observed higher levels of cytokines: TNF-α, IL-2, IL-6, IL-8, and IL-10 in severe cases [9]. Delayed adaptive immunity against COVID-19 infection is postulated to be mediated by CD4+ and CD8+ cytotoxic T cells. Lymphopenia in severe cases is attributed to the significantly lesser number of T lymphocytes. Thus, dysregulated T-cell response plays a central role in severe cases.

CRP, an inflammatory marker, is now postulated to take part in the actual inflammatory process including activation of complement pathway and phagocytosis [10]. A significant elevation of CRP in COVID-19 non-survivors would come as no surprise as hyperinflammation is the key pathology in severe cases of COVID-19.

Blood urea nitrogen (BUN), albumin, and BUN/albumin ratios were also found to be significant predictors of in-hospital mortality in a study from Turkey [11]. Even before the arrival of the COVID-19 pandemic, studies have shown that the elevation of BUN is an indicator of severity in pneumonia [12]. Now studies have emerged that BUN is a predictor of mortality in COVID-19 also [13]. Albumin is a negative acute-phase reactant, and the association of reduced albumin levels with COVID-19 severity has been observed in many studies [14].

A significant biomarker consistently shown to be a prognostic factor for predicting mortality has been D-dimer levels [15,16]. D-dimer is a known biomarker of fibrin degradation. Its elevation in COVID-19 has been postulated to be due to increased fibrin deposition with microthrombi formation and fibrinolysis, as part of COVID-19-associated coagulopathy (CAC) [17]. One of the key factors contributing to hypercoagulability in CAC is thromboinflammation whereby unregulated activation of inflammation and cytokine storm precipitates a procoagulant state [18].

Here, we present the demographic, clinical, and laboratory profiles of COVID-19-positive patients admitted in the COVID-19 wards and ICUs of a government tertiary care center in the capital city of Kerala. In the study, we compared the demographic, clinical, and laboratory profiles including D-dimer levels among survivors and non-survivors of COVID-19 infection. We also aimed to identify biomarkers of significance associated with adverse prognosis in COVID-19 patients.

The study period is during the early days of an outbreak by the SARS-CoV-2 virus before the delta variant became the predominant strain, where government hospitals were the principal provider of treatment for COVID-19-positive patients. Our center was among the major tertiary care centers in the state that stood at the forefront of the battle against the virus and catered to patients with multiple comorbidities and those referred from other designated COVID centers. The study gains significance, especially considering that studies on individual patient data from India constitute a small percentage of scientific data on COVID-19, in spite of our disease burden being the second highest in the world. Also within the country, there is high variation in social factors, the availability of health resources, prevalence of comorbidities, disease burden, and case fatality rate that warrants study from different regions. The study comes from a region with a high disease burden, prevalence of comorbidities, and a high percentage of geriatric population, but better health resources possibly translating to lower case fatality.

## Materials and methods

This is a retrospective cross-sectional study designed and conducted in Government Medical College Hospital, Thiruvananthapuram. Ethical clearance was obtained from the institutional ethical committee.

The hospital is a tertiary care and teaching hospital in the public sector, also designated for COVID-19 care. All laboratory-confirmed COVID-19-positive patients above the age of 12 years were admitted to COVID-19 wards and ICUs under the Department of Medicine. Data were collected by three investigators from the case sheets retrieved from the hospital medical records division using standardized data collection forms. Confidentiality was maintained and patient data were deidentified.

All patients admitted to COVID-19 wards and ICUs under the department of medicine with laboratory diagnosis of COVID-19 from September 21, 2020, to October 1, 2020, were included in the study. Patients who were already on anticoagulant drugs and pregnant women were excluded from the study. We collected a dataset of 391 consecutive laboratory-confirmed COVID-19-positive cases.

Demographic and clinical data including age, gender, clinical symptoms, presence of comorbidities, oxygen requirement, requirement of mechanical ventilation, ICU admission, and disease outcome were collected. Laboratory results retrieved included complete blood count, CRP, serum ferritin, lactate dehydrogenase (LDH), procalcitonin, and coagulation parameters such as D-dimer, prothrombin time international normalized ratio (PT INR), activated partial thromboplastin time (aPTT), renal function tests (RFT), and liver function tests (LFT). Initial D-dimer and peak values of D-dimer recorded during hospitalization were both included. For serum ferritin, the highest values recorded during the hospital stay were retrieved. Patients were further classified as survivors and non-survivors based on whether the patient was discharged or died. The demographic, clinical, and laboratory profiles including D-dimer levels were compared among the two groups.

Statistical analysis

Statistical analysis was performed using IBM SPSS for Windows version 25.0 (IBM Corp., Armonk, NY, USA). Continuous variables were expressed as a mean and standard deviation; categorical variables were expressed as percentages. Univariate analysis was performed to identify potential predictors of in-hospital mortality. Continuous variables were analyzed using unpaired t-test, and categorical variables using the chi-square test or Fisher’s exact test, as required. For this analysis, we prioritized the biomarker variables for model building. Initial modeling was explored for a combination of biomarkers that predicted mortality. To this realized model, we sequentially added age and comorbidities that were significant in the univariate analysis. Multivariate analysis to identify independent prognostic factors of mortality was done using stepwise backward logistic regression. Results were expressed as odds ratios (OR) with 95% confidence intervals (CI).

## Results

The data of 391 individuals were available for analysis. The mean age of all patients was 53.2 years (SD 17.0). A total of 208 patients were males and 183 were females. Breathlessness was the most common symptom with which patients presented (n = 148, 37.9%). Other symptoms in the decreasing order of frequency were fever (n = 139, 35.5%), cough (n = 106, 27.1%), myalgia (n = 38, 9.7%), sore throat (n =26, 6.6%), headache (n = 20, 5.1%), fatigue (n = 16, 4.1%), rhinitis (n = 15, 3.8%), loose stools (n = 15, 3.8 %), vomiting (n = 12, 3.1%), and anosmia (n = 4, 1.0%). A total of 125 patients were asymptomatic, which also included patients who were admitted in non-COVID wards for other conditions and were transferred to COVID-19 wards upon testing positive for RT-PCR. A total of 266 patients had at least one comorbidity (68%, 95% CI 63.3-72.5) with the most common being diabetes (n = 167, 42.7%). Other comorbidities in the decreasing order of frequency were hypertension (n = 152, 38.9%), coronary artery disease (CAD) (n = 67, 17.1%), chronic kidney disease (CKD) (n = 47, 12.0%), hypothyroid disease (n = 22, 5.6%), stroke (n = 19, 4.9%), chronic obstructive pulmonary disease (COPD) (n = 18, 4.6%), asthma (n = 17, 4.3%), chronic liver disease (CLD) (n = 17, 4.3%), malignancy (n = 15, 3.8%), and autoimmune disorder (n=1, 0.3%). Among the 391 patients admitted, the number of deaths was 60 (15.3%).

On bivariate analysis, the mean age of the survivor group was 51.1 (SD 16.7); in the non-survivor group, it was 64.7 (SD 13.4), where the difference showed statistical significance (p < 0.001). The proportion of survivors was higher among females (survivors, male: n = 172 (82.7%), female: n = 159 (86.9%); non-survivors, male: n = 36 (17.3%), female: n = 24 (13.1%), p 0.251). Diabetes, chronic renal disease, hypertension, presence of any one comorbidity, and presence of multiple comorbidities greater than two were significantly associated with mortality. The distribution of various comorbidities in the survivor and non-survivor groups is given in Table 1.

**Table 1:** Bivariate analyses of comorbidities. HTN: hypertension; COPD: chronic obstructive pulmonary disease; CAD: coronary artery disease; CKD: chronic kidney disease; CLD: chronic liver disease

Variable	Category	Survivor N=331; n (%)	Non-survivor N=60; n (%)	P-value
Presence of any one comorbidity	Yes	214 (64.7)	52 (86.7)	<0.001
	No	117 (35.3)	8 (13.3)	
Presence of diabetes	Yes	124 (37.5)	43 (71.7)	<0.001
	No	207 (62.5)	17 (28.3)	
Presence of HTN	Yes	119 (36)	33 (55)	0.005
	No	212 (64)	27 (45)	
Presence of COPD/asthma	Yes	29 (8.8)	4 (6.7)	0.591
	No	302 (91.2)	56 (93.3)	
Presence of CAD	Yes	50 (15.1)	17 (28.3)	0.120
	No	281 (84.9)	43 (71.7)	
Presence of CKD	Yes	23 (6.9)	24 (40)	<0.001
	No	308 (93.1)	36 (60)	
Presence of CLD	Yes	12 (3.6)	5 (8.3)	0.157
	No	319 (96.4)	55 (91.7)	
Presence of multiple comorbidities	Up to 2	278 (84)	30 (50)	<0.001
	3 or more	53 (16)	30 (50)	

Of all patients, 16.1% showed leukocytosis, and 36.6% and 17.6% presented with lymphopenia and thrombocytopenia (<150 × 10^9^/L), respectively. On bivariate analyses, mean values of total leukocyte count (TLC), absolute neutrophil count (ANC), and NLR were significantly higher in non-survivors than in survivors (p < 0.001). Lymphopenia was also found to be significantly associated with mortality. Neutrophilia emerged as an independent predictor of mortality on multivariate analyses.

Bivariate analysis of laboratory parameters is given in Table 2. The elevation of CRP, serum ferritin, and LDH was significant in the patients who succumbed to the disease. Although mean procalcitonin levels were found to be higher in non-survivors, no statistical significance was obtained. However, serum ferritin and CRP values were highly correlated (r = 0.53, p < 0.001). As ferritin values retrieved from the records were the highest during the hospital stay, it was excluded in the multivariate model of biomarkers. CRP (> 50mg/L) was found to be an independent factor associated with mortality, based on logistic regression analyses.

**Table 2:** Bivariate analysis of laboratory parameters. TLC: total leukocyte count; ANC: absolute neutrophil count; ALC: absolute lymphocyte count; NLR: neutrophil-to-lymphocyte ratio; PLT: platelet count; CRP: C-reactive protein; LDH: lactate dehydrogenase; PT INR: prothrombin time international normalized ratio; aPTT: activated partial thromboplastin time; SGOT: serum glutamic oxaloacetic transaminase; SGPT: serum glutamic pyruvic transaminase; A/G: albumin/globulin ratio

Variable	All patients mean (SD)	Survivor mean (SD)	Non-survivor mean (SD)	P-value
TLC (×10^9^/L)	8.2 (5.2)	7.5 (4.2)	11.9 (7.6)	<0.001
ANC (×10^9^/L)	6.1 (5.1)	5.3 (4.1)	10.5 (7.4)	<0.001
ALC (×10^9^/L)	1.9 (1.0)	2.0 (0.9)	1.3 (1.0)	<0.001
NLR	4.7 (6.9)	3.4 (3.5)	11.6 (13.5)	<0.001
PLT (×10^9^/L)	220 (90)	220 (80)	230 (110)	0.644
CRP (mg/L)	70 (104)	48 (85)	185 (117)	<0.001
Ferritin (ng/ml)	404.8 (447.2)	323.6 (374.1)	829.4 (551.2)	<0.001
LDH (U/L)	563.2 (438.2)	485.1 (353.9)	905.5 (589.1)	<0.001
Procalcitonin (ng/ml)	0.7 (0.8)	0.45 (0.68) n = 19	0.86 (0.91) n = 22	0.112
D-dimer at admission (µg/ml)	1.7 (2.5)	1.29 (2.08)	4.01(3.53)	<0.001
D-dimer highest (µg/ml)	2.1 (3.2)	1.50 (2.38)	5.73 (4.56)	<0.001
PT INR	1.0 (0.3)	0.99 (0.18)	1.21 (0.42)	0.001
aPTT (sec)	34.7 (48.8)	35.5 (58.3)	32.9 (9.2)	0.770
Urea (mg/dl)	44.3 (51.9)	33.6 (31.0)	105.1 (91.4)	<0.001
Creatinine (mg/dl)	1.5( 2.4)	1.1 (1.6)	3.6 (4.1)	<0.001
Total bilirubin (mg/dl)	0.8 (1.3)	0.75 (1.42)	0.76 (0.67)	0.976
Direct bilirubin (mg/dl)	0.2 (0.6)	0.22 (0.67)	0.24 (0.30)	0.796
SGOT (U/L)	44.6 (52.1)	44.6 (55.3)	44.8 (28.6)	0.984
SGPT (U/L)	44.5 (59.2)	46.4 (63.3)	33.6 (23.4)	0.302
Total protein (g/dl)	6.9 (3.0)	7.0(3.2)	6.3 (0.8)	0.148
Albumin (g/dl)	3.6 (1.9)	3.8 (2.1)	3.0 (0.7)	0.005
A/G	1.2 (0.4)	1.2 (0.4)	0.9 (0.3)	<0.001
Alkaline phosphatase (U/L)	87.8 (96.5)	79.4 (62.0)	136.3 (196.4)	0.043

The mean value of D-dimer at admission was 1.7 µg/ml. The mean value at admission was 4.0 µg/ml in the non-survivors and 1.29 µg/ml in the survivors (p<0.001). Similarly, the highest in-hospital D-dimer values were also significantly more elevated in the non-survivors. The mean PT INR value was 1.21 in the non-survivors and 0.99 in the survivors (p < 0.001). D-dimer at admission was found to be associated with mortality on logistic regression model analyses.

Increased BUN and creatinine as well as low albumin and albumin/globulin (A/G) ratio in the non-survivors were found to be statistically significant. Of these, BUN remained as an independent predictor of mortality in the multivariate analysis. When the model was repeated after removing 47 patients who already had CKD at admission, all predictors except CRP continued to be significantly associated with adverse outcomes.

We did not get any other significant predictors with age and other comorbidities in any of the sequential models. The regression model that we ultimately report here is based on a sample size of 265, for whom complete biomarkers were available. This is effectively a coverage of 67.8% of our sample space. Our study was adequately powered to conduct this analysis (84.9%). Multivariate analysis of selected variables is given in Table 3.

**Table 3:** Multivariate analysis on selected parameters. *p < 0.05, **p < 0.01, ***p < 0.001 ANC: absolute neutrophil count; CRP: C-reactive protein; BUN: blood urea nitrogen Laboratory reference values: CRP (quantitative): 0-50 mg/L; D-dimer (immunotubidimetric): 0-0.5 µg/ml; BUN (Dry chemistry): 15-40 mg/dl

Variable	Category	Crude OR (95% CI)	Adjusted OR (95% CI)
ANC (×10^9^/L)	≤7.0	1.00	1.00
	>7.0	6.16 (3.38–11.22)	2.81* (1.18–6.70)
CRP (mg/L)	≤50	1.00	1.00
	>50	13.72 (6.29–29.96)	3.62* (1.35–9.74)
D-dimer at admission (µg/ml)	<0.5	1.00	1.00
	0.5–1.0	3.15 (0.56–17.63)	1.27 (0.18–8.97)
	>1.0	31.54 (7.41–134.21)	7.94* (1.64–38.58)
BUN (mg/dl)	≤40	1.00	1.00
	>40	12.15 (6.23–23.69)	4.42** (1.81–10.82)

## Discussion

The mean age of all patients was 53.2 years (SD 17.0). Males outnumbered females and accounted for 53.2% of the patients. Breathlessness (37.9%), cough (35.5%), and fever (27.1%) were the most common symptoms reported at the time of admission. The less common symptoms were myalgia, sore throat, headache, fatigue, rhinitis, loose stools, vomiting, and anosmia. More patients reported complaints of breathlessness along with fever and cough, compared to other studies [19,20]. The probable reason could be that ours being a referral center, many patients might have been admitted days after the onset of disease. The leading comorbidity in our study population was diabetes (42.7%) followed by hypertension (38.9%), CAD (17.1%), and CKD (12.0%). Around 68% of the patients had at least one comorbidity. In a study conducted by Guan et al., where 1099 patients were analyzed, the proportion of patients with at least one comorbidity was 23.7% [6]. A study from Medical College Hospital in Jaipur showed the comorbidity rate as 13.98%, but their patient mean age was only 35.42 years [20]. Compared to studies from China and from other Indian states, the proportion of comorbidity in our patient population was high. Two factors could account for this: (1) the baseline high prevalence of comorbidities especially diabetes in the state of Kerala, and (2) being a tertiary care center, our institute receives many of the high risk and seriously ill patient referrals from other hospitals. Of the 391 patients, the proportion of death was 15.3% (95% CI 12.1-19.2). This is in line with the higher in-hospital case fatality rate in referral tertiary care centers compared to the general population. A tertiary care medical center in the USA, which also had a high proportion of patients with comorbidities (95.2%), reported a case fatality rate of 30% during the early phase of the pandemic [19].

In bivariate analysis, age, presence of diabetes, CKD, hypertension, and multiple comorbidities of more than two were also significantly associated with mortality. These findings are comparable with the various studies that identified age, the presence of diabetes, and CKD as significant risk factors for mortality in COVID-19 [21-23].

Of the patients with COVID-19 admitted during the study period, 36.6% and 17.6% presented with lymphopenia and thrombocytopenia, respectively. In comparison, a study by Guan et al. found that 83.2% of patients had lymphopenia and 36.2% thrombocytopenia at the time of admission. In bivariate analyses, the mean values of TLC, ANC, and NLR were significantly higher in non-survivors than in survivors. However, the absolute lymphocyte count was significantly lower in non-survivors. Neutrophilia was found to be an independent predictor of adverse outcomes. Several studies have observed neutrophilia, increased NLR, and lymphopenia to be associated with mortality [5-7]. A meta-analysis by Huang and Pranata on 24 studies with 3099 patients showed an association between lower lymphocyte counts and those who died, those who experienced ARDS, and those who received ICU care: mean difference − 395.35 μL − 165.64, − 625.07), p < 0.001, I^2^ 87%; mean difference − 377.56 μL (− 271.89, − 483.22), p < 0.001; I^2^ 0%; mean difference − 376.53 μL (− 682.84, − 70.22), p = 0.02, I^2 ^89%, respectively [24]. In a retrospective study on 93 patients, elevated NLR (HR 2.46, 95% CI 1.98-4.57) was observed to be an independent risk factor for adverse clinical outcomes of COVID-19 [7]. Wu et al. reported that neutrophilia was associated with an increased risk of developing ARDS and further progression to death (HR 1.14; 95% CI 1.09-1.19 and HR 1.08; 95% CI 1.01-1.17, respectively) [15].

Hyperactivation of neutrophils along with macrophages is postulated to stimulate the release of proinflammatory cytokines, setting off the cytokine storm. Similarly, dysregulation of T-lymphocyte response, which is an integral part of the delayed adaptive immune response, plays an important role in severe cases. Therefore, these simple and accessible hematological tests can be used in picking up severe cases in low-resource settings.

The platelet count did not show any significant difference between the two groups. Activation of liver with thrombopoietin release could be the reason for the relatively normal platelet counts reported in COVID-19 infections [25]. However, platelet count at admission was collected during the study. A fall in the platelet count during the hospital stay was noted in some patients. It would be worthwhile to study the dynamic changes in platelet counts in COVID-19 patients during their hospital stay.

Among the inflammatory markers, CRP, serum ferritin, and LDH were positively associated with mortality. With logistic regression analysis, CRP (>50mg/L) was found to be an independent predictor of mortality, which corroborates with the outcomes of previous studies [6,15,26,27]. Even earlier, CRP has been suggested as an early marker to predict in-hospital mortality. A Swedish multicenter study showed that an admission CRP level above 100 mg/L was a predictive factor for increased ICU rate and increased 30-day mortality [28]. Some of our patients showed an increase in ferritin levels during their hospital stay, with values going up from within normal limits to above 2000 IU/L, especially in non-survivors. Hence, the highest values and not the values at admission were collected for serum ferritin, which was found to be statistically significant. For the same reason, ferritin was excluded from the multivariate analyses. A study with serial measurement of serum ferritin levels is worth exploring. Low ferritin levels were also observed in a few of our patients, possibly as part of iron deficiency.

The mean D-dimer of all patients at admission was above normal limits. The mean D-dimer levels at admission and the highest D-dimer value in non-survivors were significantly elevated (mean SD survivor vs non-survivor 1.29 (2.08) vs 4.01 (3.53), 1.50 (2.38) vs 5.73 (4.56) (µg/ml); p < 0.001). Similarly, PT INR was also significantly increased in patients with adverse outcome (mean SD 0.99 (0.18) vs 1.21 (0.42); p < 0.001). Among the two coagulation parameters, only D-dimer at admission (>1 µg/ml) remained as a variable of significance in logistic regression model analyses. This is similar to multiple studies from China that have shown raised D-dimer levels to be a prognostic marker of disease severity and mortality [6,29,30]. Generally, in CAC, elevation in PT is limited to a few patients and only mildly prolonged compared to DIC [31]. However, a significant elevation of PT INR in non-survivors seen in our patients was akin to the findings in the study from Faridabad, North India [32]. Similar studies in the local population, in the future, would help to elucidate the coagulation profile of the Indian population in CAC.

Increased BUN, creatinine, alkaline phosphatase, and low serum albumin, as well as A/G ratio in non-survivors, were found to be statistically significant. Of these, BUN (>40mg/dl) remained as an independent predictor of mortality in logistic regression analysis. Even when patients with CKD were removed and the model repeated, BUN continued to be an independent predictor of adverse outcomes.

The elevation of BUN independent of creatinine reflects hypoperfusion of the kidney secondary to hypovolemia, sepsis, or decreased cardiac output [33,34]. A multicenter observational study conducted in intensive care units of two teaching hospitals in Boston identified BUN as an independent predictor of all-cause mortality [35].

Limitations

The retrospective design of the study may bring along inherent biases associated with it. Ours being a referral tertiary care center, catering to predominantly severely ill patients, might have tilted the analyses toward null. In addition, some parameters like procalcitonin were investigated only in a few patients and could not be taken up for further analysis, which might have introduced bias to these analyses.

## Conclusions

In conclusion, our study identified several clinical and laboratory factors that showed an association with COVID-19-related mortality. These include comorbidities like diabetes, hypertension, and CKD; hematological variations like leukocytosis, neutrophilia, increased NLR, and lymphopenia; coagulation parameters like D-dimer levels at admission and PT INR; inflammatory markers like CRP, peak ferritin levels, and LDH; and also BUN, low serum albumin, and A/G ratio. Among these, neutrophilia, CRP, D-dimer at admission, and BUN emerged as independent predictors of mortality. Many of these are simple and accessible tests that can be implemented even in low-resource settings that can help in the rational use of scarce resources during a pandemic. Additionally, based on this work, studies to assess dynamic changes in platelet count and ferritin levels during hospital stays are recommended.

## References

[1] Zhu N, Zhang D, Wang W (2020). A novel coronavirus from patients with pneumonia in China, 2019. N Engl J Med.

[2] World Health Organization. WHO Director-General’s opening remarks at the media briefing on COVID-19. https://www.who.int/director-general/speeches/detail/who-director-general-s-opening-remarks-at-the-media-briefing-on-covid-19---11-march-2020.

[3] Andrews MA, Areekal B, Rajesh KR (2020). First confirmed case of COVID-19 infection in India: A case report. Indian J Med Res.

[4] Rajan SI, Shajan A, Sunitha S (2020). Ageing and elderly care in Kerala. China Rep.

[5] Zhao Q, Meng M, Kumar R (2020). Lymphopenia is associated with severe coronavirus disease 2019 (COVID-19) infections: A systemic review and meta-analysis. Int J Infect Dis.

[6] Guan WJ, Ni ZY, Hu Y (2020). Clinical characteristics of coronavirus disease 2019 in China. N Engl J Med.

[7] Yang AP, Liu JP, Tao WQ, Li HM (2020). The diagnostic and predictive role of NLR, d-NLR and PLR in COVID-19 patients. Int Immunopharmacol.

[8] Chan L, Karimi N, Morovati S (2021). The roles of neutrophils in cytokine storms. Viruses.

[9] Qin C, Zhou L, Hu Z (2020). Dysregulation of immune response in patients with Coronavirus 2019 (COVID-19) in Wuhan, China. Clin Infect Dis.

[10] Thiele JR, Habersberger J, Braig D (2014). Dissociation of pentameric to monomeric C-reactive protein localizes and aggravates inflammation: in vivo proof of a powerful proinflammatory mechanism and a new anti-inflammatory strategy. Circulation.

[11] Küçükceran K, Ayrancı MK, Girişgin AS, Koçak S, Dündar ZD (2021). The role of the BUN/albumin ratio in predicting mortality in COVID-19 patients in the emergency department. Am J Emerg Med.

[12] Lim WS, van der Eerden MM, Laing R (2003). Defining community acquired pneumonia severity on presentation to hospital: an international derivation and validation study. Thorax.

[13] Cheng A, Hu L, Wang Y (2020). Diagnostic performance of initial blood urea nitrogen combined with D-dimer levels for predicting in-hospital mortality in COVID-19 patients. Int J Antimicrob Agents.

[14] Viana-Llamas MC, Arroyo-Espliguero R, Silva-Obregón JA (2021). Hypoalbuminemia on admission in COVID-19 infection: An early predictor of mortality and adverse events. A retrospective observational study. Med Clin (Barc).

[15] Wu C, Chen X, Cai Y (2020). Risk Factors associated with acute respiratory distress syndrome and death in patients with coronavirus disease 2019 pneumonia in Wuhan, China. JAMA Intern Med.

[16] Zhou F, Yu T, Du R (2020). Clinical course and risk factors for mortality of adult inpatients with COVID-19 in Wuhan, China: a retrospective cohort study. Lancet.

[17] Adam SS, Key NS, Greenberg CS (2009). D-dimer antigen: current concepts and future prospects. Blood.

[18] Sarkar M, Madabhavi IV, Quy PN, Govindagoudar MB (2021). COVID-19 and coagulopathy. Clin Respir J.

[19] Bhargava A, Szpunar SM, Sharma M (2021). Clinical features and risk factors for in-hospital mortality from COVID-19 infection at a tertiary care medical center, at the onset of the US COVID-19 pandemic. J Intensive Care Med.

[20] Bhandari S, Singh A, Sharma R (2020). Characteristics, treatment outcomes and role of hydroxychloroquine among 522 COVID-19 hospitalized patients in Jaipur City: An epidemio-clinical study. J Assoc Physicians India.

[21] Ho FK, Petermann-Rocha F, Gray SR (2020). Is older age associated with COVID-19 mortality in the absence of other risk factors? General population cohort study of 470,034 participants. PLoS One.

[22] Huang I, Lim MA, Pranata R (2020). Diabetes mellitus is associated with increased mortality and severity of disease in COVID-19 pneumonia - A systematic review, meta-analysis, and meta-regression. Diabetes Metab Syndr.

[23] Gansevoort RT, Hilbrands LB (2020). CKD is a key risk factor for COVID-19 mortality. Nat Rev Nephrol.

[24] Huang I, Pranata R (2020). Lymphopenia in severe coronavirus disease-2019 (COVID-19): systematic review and meta-analysis. J Intensive Care.

[25] Thachil J (2020). What do monitoring platelet counts in COVID-19 teach us?. J Thromb Haemost.

[26] Ali N (2020). Elevated level of C-reactive protein may be an early marker to predict risk for severity of COVID-19. J Med Virol.

[27] Luo X, Zhou W, Yan X (2020). Prognostic Value of C-Reactive Protein in Patients With Coronavirus 2019. Clin Infect Dis.

[28] Koozi H, Lengquist M, Frigyesi A (2020). C-reactive protein as a prognostic factor in intensive care admissions for sepsis: A Swedish multicenter study. J Crit Care.

[29] Tang N, Li D, Wang X, Sun Z (2020). Abnormal coagulation parameters are associated with poor prognosis in patients with novel coronavirus pneumonia. J Thromb Haemost.

[30] Yao Y, Cao J, Wang Q (2020). D-dimer as a biomarker for disease severity and mortality in COVID-19 patients: a case control study. J Intensive Care.

[31] Connors JM, Levy JH (2020). COVID-19 and its implications for thrombosis and anticoagulation. Blood.

[32] Raychaudhuri S, Pujani M, Menia R (2021). COVID-19 Associated Coagulopathy in an Indian Scenario: A Correlation with Disease Severity and Survival Status. Indian J Hematol Blood Transfus.

[33] Dal Canton A, Fuiano G, Conte G, Terribile M, Sabbatini M, Cianciaruso B, Andreucci VE (1985). Mechanism of increased plasma urea after diuretic therapy in uraemic patients. Clin Sci (Lond).

[34] Aronson D, Mittleman MA, Burger AJ (2004). Elevated blood urea nitrogen level as a predictor of mortality in patients admitted for decompensated heart failure. Am J Med.

[35] Beier K, Eppanapally S, Bazick HS, Chang D, Mahadevappa K, Gibbons FK, Christopher KB (2011). Elevation of blood urea nitrogen is predictive of long-term mortality in critically ill patients independent of "normal" creatinine. Crit Care Med.

